# Performance of urban storm drainage network under changing climate scenarios: Flood mitigation in Indian coastal city

**DOI:** 10.1038/s41598-019-43859-3

**Published:** 2019-05-23

**Authors:** Ramachandran Andimuthu, Palanivelu Kandasamy, B V Mudgal, Anushiya Jeganathan, Abinaya Balu, Guganesh Sankar

**Affiliations:** 1 Centre for Climate Change and Adaptation Research, Department of Civil Engineering, CEG Campus, Anna University, Chennai, 600025 India; 2 Centre for Water Resources, Department of Civil Engineering, CEG Campus, Anna University, Chennai, 600025 India

**Keywords:** Climate sciences, Environmental sciences, Hydrology

## Abstract

Managing storm water under climate uncertainty is a major concern in urban areas throughout the world. There were several floods events recorded in Chennai, a one of the major metropolitan coastal city in India. The flood incidences were repeatedly reported in recent decades. In this study, the existing state of storm water drains are evaluated under current and future climate scenarios in one of the most flood-prone areas of Chennai viz. Velachery zone. The mitigation measures are recommended to increase its resilience against floods. The Intergovernmental Panel on Climate Change (IPCC) CMIP5 models of Representative Concentration Pathways (RCP) 4.5 are used to develop possible future climate change scenarios of the city. The daily rainfall data for the period 1975–2015 obtained from India Meteorological Department are used to find the extremities and to generate Intensity-Duration-Frequency (IDF) curves. The IDF curves are generated for 2, 5, 10, 50, 100 year return period under current and future climate scenarios. The storm drainage network are delineated with Differential Geographic Positioning System (DGPS) survey. The integrated hydraulic and hydrological modelling is carried out to assess the flood carrying capacity of storm drainage under present and future climate scenarios. The vulnerable hotspots are identified and flood mitigation measures are suggested to reduce the flood risk at Velachery.

## Introduction

Nearly 54% of world’s population lives in urban areas and it is expected to increase up to 66% by 2050 and most of these urban areas are coastal cities. The 2014 revision of the World Urbanization Prospects by UN DESA’s Population Division says that the huge rate of urban growth will take place especially in India, China and Nigeria^1^. While, these urban systems are most critical structures in modern societies under changing climate scenarios, have disruptions and significant effects on the daily life of urban inhabitants^2^. As per the Intergovernmental Panel on Climate Change (IPCC) Assessment Report 5, the number of heavy precipitation events has increased on land regions. In the Asian region, strong variability is observed in precipitation trends and extremes in different parts and seasons of Asia^3^. This changes in climate will disproportionately affect cities mostly located in climate-sensitive areas such as floodplains and coastal zones. The most important effects of climate change on cities are likely to be on water availability, flooding (pluvial, fluvial and coastal) from heavy precipitation, increased surface and stream erosion and overloading of storm water treatment systems during high flow events. In typical urban storm water networks, vulnerabilities such as flooding arise as the capacity of components within the system is overloaded and runoff accumulates at the surface. Studies to date indicate that the response of drainage systems to projected climate changes is site dependent^4,5^. These conditions have implications for site-specific storm water and floodplain management as local decision makers look to improve existing infrastructure and build new storm water systems. There is an increased attention on urban storm water management and flood mitigation in vulnerable regions especially in developing countries such as India. The recent climate impact assessment studies provide insights into how climate change information can be incorporated into local planning and decision making through an integrated approach for sustainable urban water management^6–8^.

Recent extreme rainfall events resulting in flooding across many of the Indian cities have forced governments to initiate actions towards the impact of climate change on local conditions and adaptation/mitigation actions that can fit into decision-making processes. Being a coastal city, Chennai faces flood threats often both through cyclonic activities and monsoon rains. In the past, several flood events were recorded at Chennai and the flood incidences were repeatedly reported in the recent decades during 2002, 2005, 2006, 2007, 2008, 2010, 2015 and 2016. Chennai was severely flooded due to heavy rains (16–20 cm) during October 30 to November 2, 2002. Residential areas became ‘islands’ and were cut-off, causing disruption in services and trade, including transport, communication, etc. A deep depression over the Bay of Bengal brought about 42 cm rainfall within a 40 h duration during the North-East monsoon of 2005^9^. During the flood in 2008 which was of moderate intensity, residential areas near Taramani link road got submerged in rain water. During November and December 2015 Chennai witnessed heavy rainfall incidence that took away many lives and caused water logging in many parts of the city. The severity of flood was extreme and the financial cost of the flood was unprecedented. The entire commercial, trading, transport (both land and air) and industrial activity stopped for several days. These extreme events stress the need for flood assessment in Chennai under future climate change scenarios. In 2016, cyclone ‘Vardah’ brings heavy rainfall in Chennai and many area were inundated.

**Figure 1:**
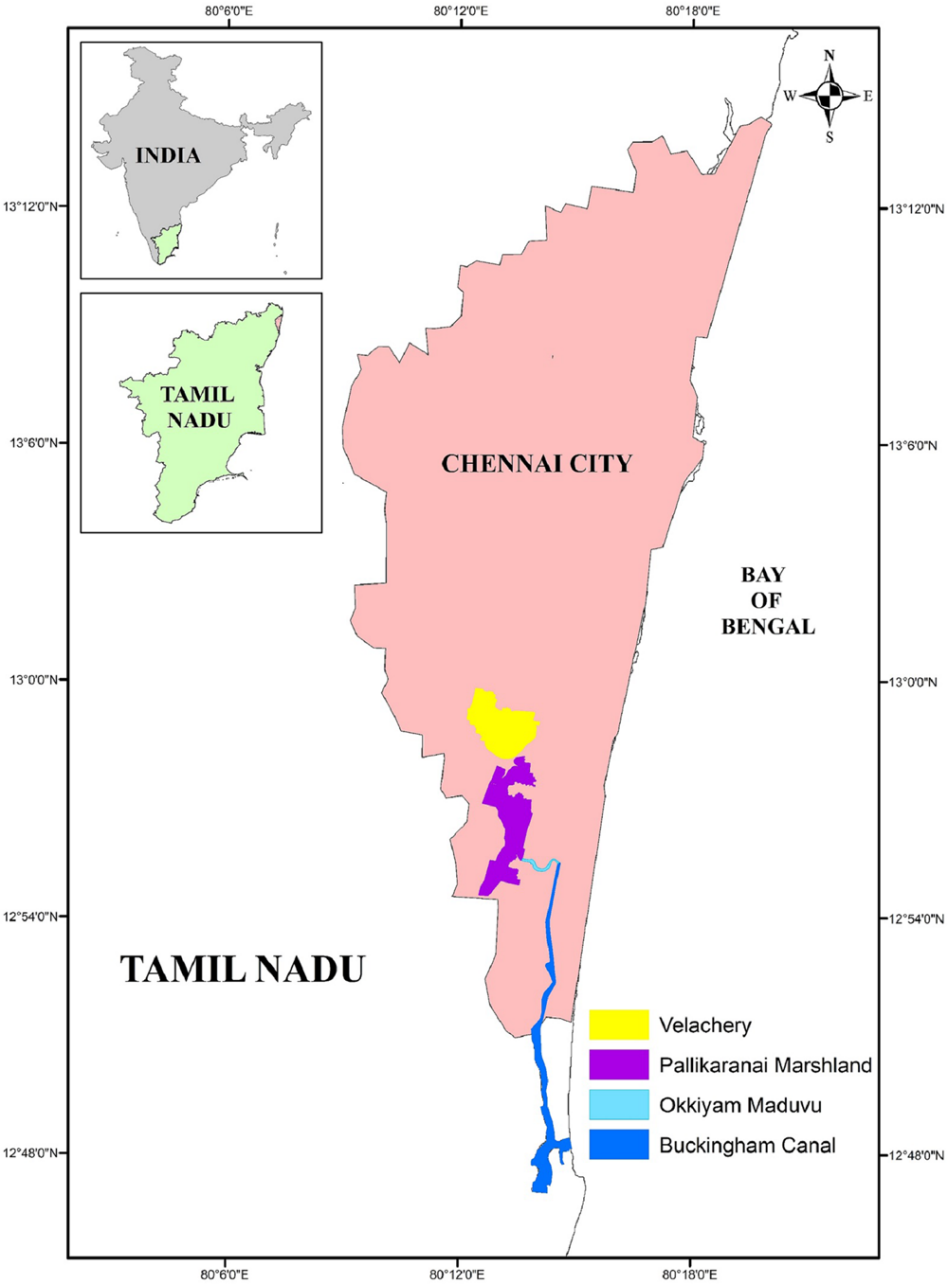
Location map of the study area.

Recent heavy flooding events in Chennai have prompted to have a relook into existing state of storm water drains stresses the need for flood management studies under present and future climate scenarios. The study area Velachery in Chennai Metropolitan Area is frequently affected by flood resulting in water stagnation. The present study addresses the impacts of climate change on storm water management at Velachery – a flood-prone area of Chennai. Velachery, a rapidly growing zone of southern Chennai Metropolitan Area lies between Latitudes 12°58′20″N and 12°58′33″N and Longitudes 80°13′35″E and 80°13′17″E. The boom in Information Technology during the last decade has accelerated the growth of Velachery into a commercial and residential hub. Rapid urbanization is continuously encroaching on the drainage system of Velachery. This area is known for frequent inundation, resulting from storm water stagnation and choking of storm drain network. The storm water from Velachery passes through the Pallikaranai marshland and reaches Okkiyam Maduvu channel. Storm water from Okkiyam Maduvu then passes through Buckingham Canal and reaches the sea at Muttukadu, Kovalam. Okkiyam Maduvu is a 2.8 kilometres long water channel originates as a narrow canal from the Pallikaranai marshland and drains into the Buckingham canal which flows south and enters the Kovalam estuary. The location of the study area is shown in Fig. 1. Thus, this work is carried out to evaluate the storm water drain performance under climate change scenarios and to identify the vulnerable hot spots in Velachery Zone & propose mitigation measure for free flow of the storm water. The scientific knowledge of the study will help city planners to design intelligent climate-proof urban storm water drainage design.

## Results and Discussion

### Observed rainfall extreme events and projected future climate scenario

**Figure 2:**
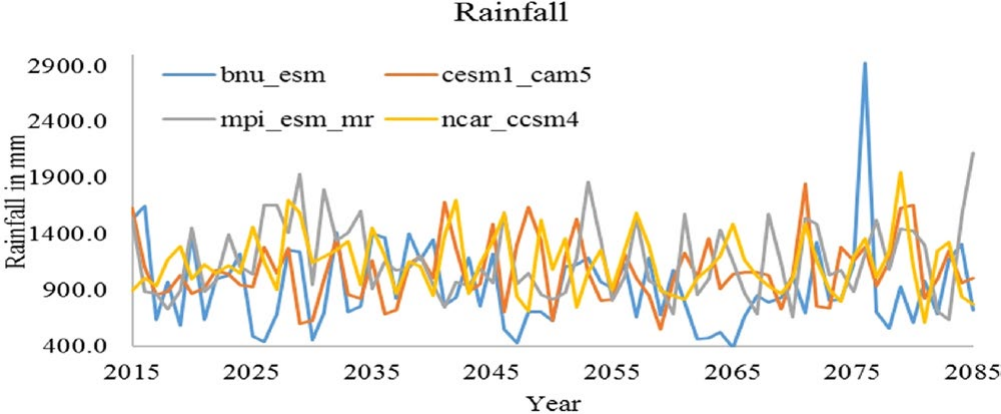
Rainfall projections with GCM models till 2085.

The rainfall extremities are one of the major concerns of changing the climate. The occurrences of extreme events in the study area were calculated based on Indian Meteorological Department norms. The extremely heavy rainfall event (>244.5 mm/day) and very heavy rainfall event (>124.5 mm/day) for the period of 1975–2015 were calculated. The results show that the occurrence of the extremely heavy rainfall events and very heavy rainfall events are increasing in recent years. Out of 46 occurrences of very rainfall events, 15 events recorded within the period 2000–2015. Eight extreme heavy rainfall events occurred during 1975–2015. In 1976 extremely heavy rainfall event was recorded with the intensity of 346.6 mm/day and 345.1 mm/day was recorded in 2015. Rainfall data from the 4 GCM (Global Climate Models) models for the period 2015–2085 were estimated to project the future rainfall trends are shown in Fig. 2.

### Design storm

**Figure 3:**
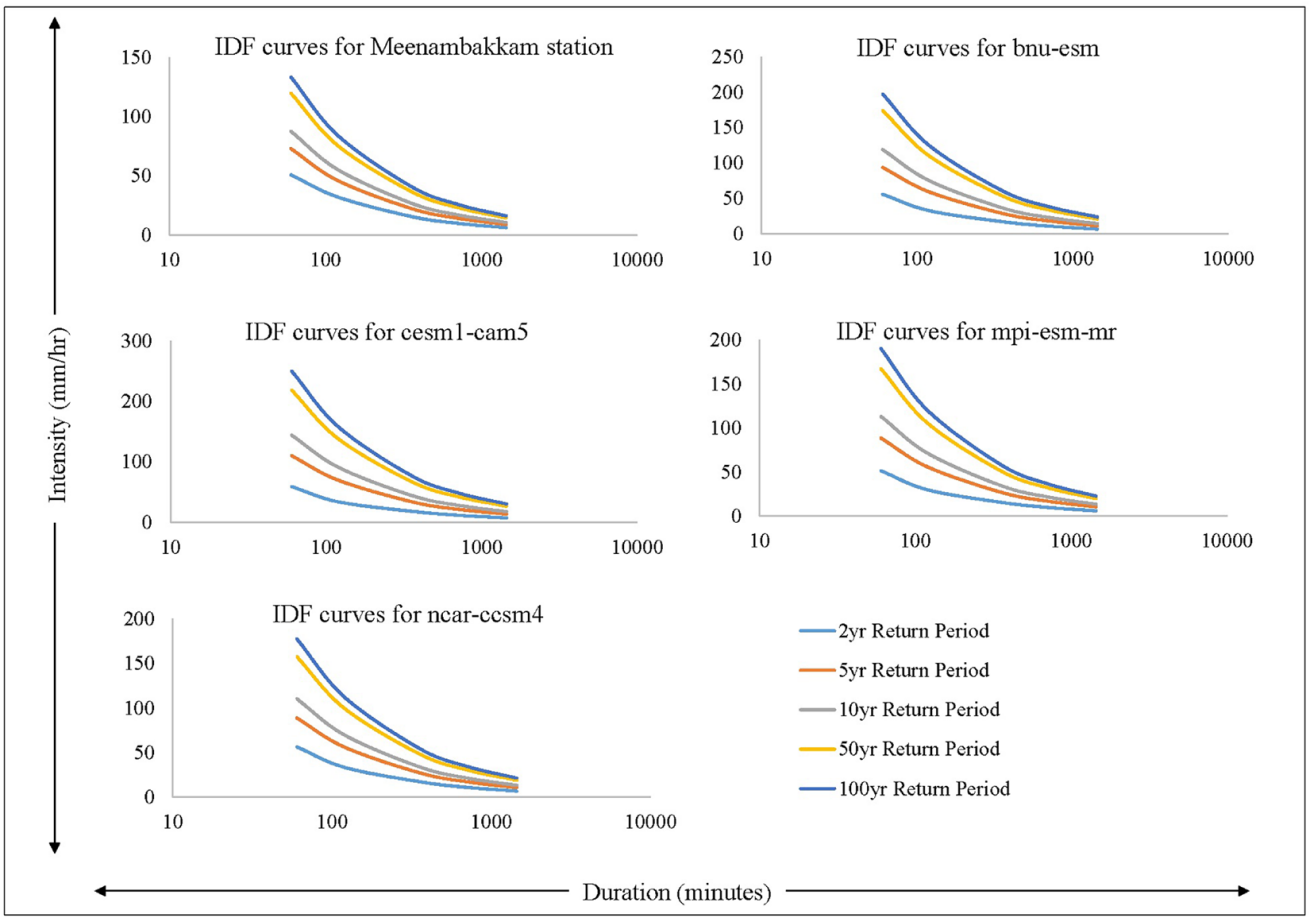
IDF curves for observed climate data and selected climate models.

Design storms were estimated for different return periods under past and future scenarios. IDF (Intensity Duration Frequency) curves were developed for observed (historical climate) and 4 GCMs of RCP 4.5 pathway (projected climate scenarios) (Fig. 3). IDF curves were developed for 5 different durations (1 h, 2 h, 6 h, 12 h and 24 h) with 5 return periods of 2, 5, 10, 50, 100 years for observed and projceted climate scenario. Therefore, 25 annual maximum time series were used for the frequency analysis to develop the IDF curves. The result shows that the intensity of rainfall for 2 year return period is 33.57 mm/hr under observed climate. Similarly, for 10 year return period, the intensity is 57.89 mm/hr and 88.22 mm/hr intensity is estimated for 100 year return period rainfall. Intensities for 2 year return period for models cesm1_cam5, mpi_esm_mr, ncar_ccsm4, bnu_esm are 37.61 mm/hr, 33.32 mm/hr, 36.50 mm/hr and 36.03 mm/hr respectively. Intensities for 100 year return periods are 165.67 mm/hr, 126.6 mm/hr, 117.75 mm/hr and 130.79 mm/hr. These estimated time series were used in the model for the simulation process.

### Hydrologic modelling

**Figure 4:**
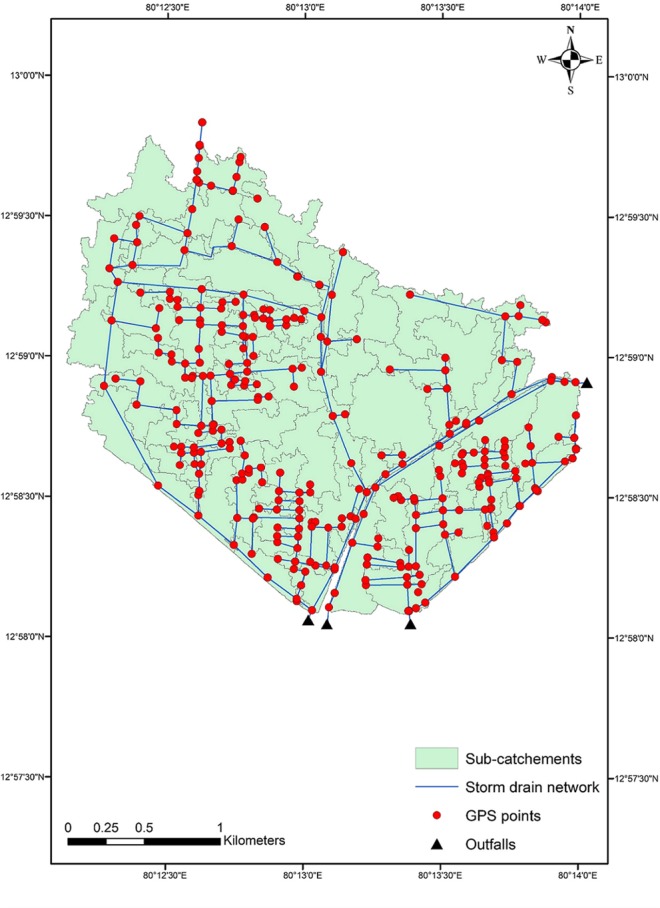
Storm drainage network of study area.

Delineation of the sub-catchment was done by using ArcSWAT in ArcGIS software (Fig. 4). The total watershed area is 697.1 ha and further delineated to 121 smaller sub-catchments. The parameter details like area, width, imperviousness rate and average slope were derived from these sub-catchments. Nearly 88.5% area is impervious and the elevation ranges from 1.87 m to 13.2 m. Performance of the storm drain in the study area was analyzed by simulating the model for rainfall of 5 different return periods as (2 yr, 5 yr, 10 yr, 50 yr and 100 yr). Likewise, the model was used to simulate for future projected rainfall data. All the inputs except design storm were same for all the simulations. The simulation results on rainfall, infiltration, runoff and inflow/outflow for the conveyance system show that most of the rainfall in the study area is converted into a surface runoff. For 2 yr return period the simulated runoff is 32.402 mm for a given rainfall of 33.567 mm. Infiltration loss is only 0.455 mm due to the imperviousness of the study area. Similarly from 88.223 mm rainfall, 87.292 mm is converted into a surface runoff for 100 yr return period. Final surface storage is ranged from 0.847 to 0.894 mm for 2 to 100 yr return period. The result shows that infiltration is very less due to high imperviousness of the study area. Surface storage also very less due to flat terrain. Most of the rainfall results into surface runoff because the capacity of the storm drains is inadequate for intense rainfall and flooding takes place.

Outfall 1 is the major outlet. This outlet is located near Velacherry railway station and joins with Pallikaranai marshland. Most of the storm drains in the study area are connected to outfall 1. (Fig. 4). The model predicts a total peak discharge as 13.918 m^3^/sec for 2 year return period rainfall and 52% of the total peak discharge is from outfall 1 as 7.29 m^3^/sec. Total peak discharge is recorded as 21.9 m^3^/sec for 100 year return period rainfall at outfall 1 which is 64% in total peak discharge from the study area. Out of 4 outfalls, outfall 1, 2 and 4 joins with the Pallikaranai marshland and outfall 3 is connected with Buckingham canal. The maximum flooding occurred at junction VB-179 which was located in 100 ft road very near to Velachery lake and the flow rate was 36.554 m^3^/s. Nearly 15 nodes were flooded for many hours for even a 2 year return period rainfall. There was little inflow in 48 nodes due to the elevation change. It shows that the existing storm drains are poorly designed and constructed. Reconstruction of the drains according to the flow routing of that area will reduce the flood risk and will minimize the flooding duration.

**Figure 5:**
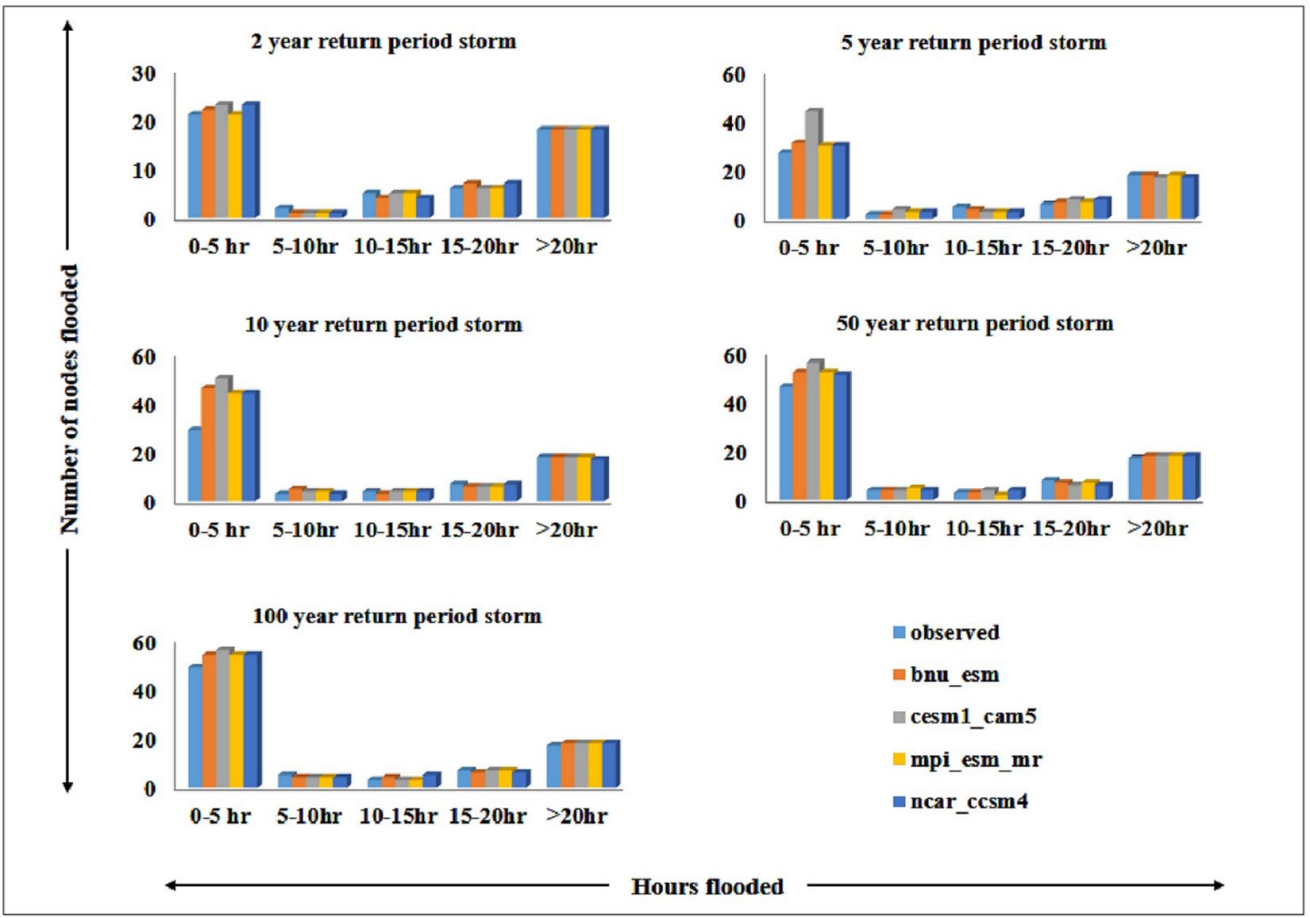
Number of nodes flooded under 2, 5, 10, 50 and 100 year return period storms for observed and projected climate.

Figure 5 shows the number of nodes flooded for different return periods under observed and projected climate scenarios. Duration of flooding were categorized as 0–5 h, 5–10 h, 10–15 h, 15–20 h and >20 h. A number of nodes flooded for different return periods of observed climate were compared with GCMs models results. The number of nodes flooded are increased with the increase in return period. Nearly 23 nodes were flooded for about 5 hours for a 2 year return period and nearly 50 nodes were flooded for the 100 year return period. Some of the nodes idenfied flooding nearly 1 day for all the return period under observed and projected climate scenarios. These vulnerable nodes were located near Taramani link road connecting with Velachery main road towards Tambaram. During rainy days these places have been noticed severely affected by floods for many hours and sometimes for days. Inundated water stagnation on the roads create a heavy traffic jam and also spread waterborne diseases.

The result clearly shows that the existing storm drain network cannot withstand even for a 2 year return period rainfall. In some places, the inlets of the storm drains are either blocked with debris or blocked by elevated roads. Due to this, the surface runoff could neither enter nor freely flow into the storm drains. The inadequacy of the storm drains along with poor maintenance increases the duration of ponding.

**Figure 6:**
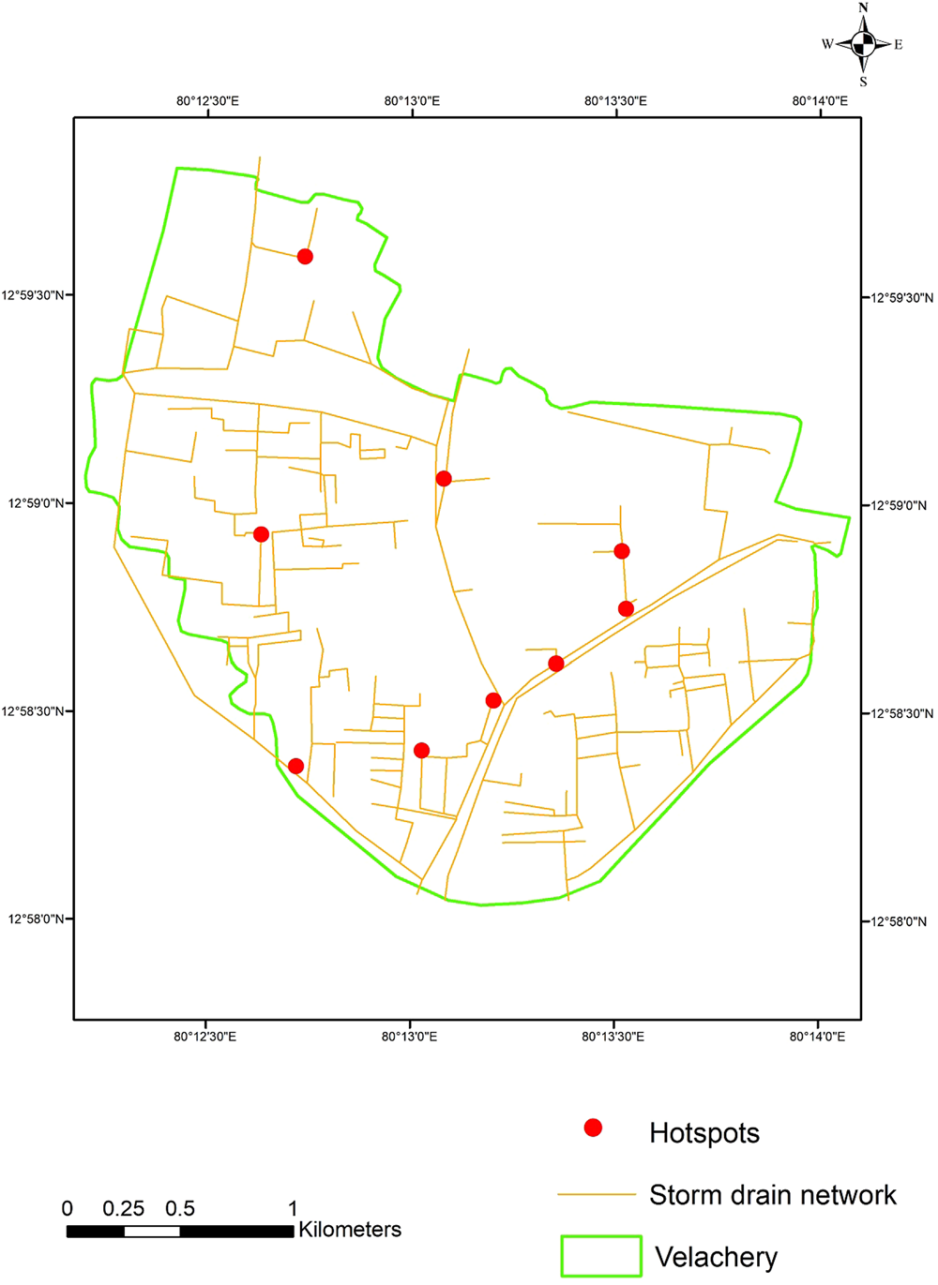
Vulnerable hotspots of the study area.

Among conduits, L32B-179, L23–177, Loe-178, Lof-178, Lod-178, out-178, out-179 attained maximum/full depth. Based on the observed condition, 84 conduits attained full depth for 2 year return period. It may increase up to 88 conduits under projected cliamte scenario. For 100 year return period, 111 conduits surcharged under the observed condition and 121 for projected future climate scenario. The number of nodes that surcharge are 51, 58, 60, 78 and 81 for rainfall of return periods 2 yr, 5 yr, 10 yr, 50 yr and 100 yr respectively. Many nodes are noticed with more than 20 hours of flooding. The areas near 100 ft road (near Velachery lake), LIC colony 2^nd^ street, Dhandeeswaram 7^th^ avenue east, Dhandeeswaram 7^th^ main road, Southern arm of Inner Ring road, Vijaya Nagar 7^th^ main road, T.N.H.B 3^rd^ main road, Srinagar colony main road, Nethaji colony are identified as hot spots and experienced flooding under all storm events. The hot-spots area prone to flooding are shown in Fig. 6.

### Mitigation option to reduce the flood vulnerability in Velachery

The rainfall-runoff model simulation was carried out in HEC-HMS. The model simulated a peak discharge of 1349.5 m^3^/s in Okkiyam Maduvu for 2015 flood (165 yr return period rainfall). The width of Okkiyam Maduvu weir is measured as 120 m. From the weir equation, the height of water above the crest of the weir is 3.43 m for the peak discharge. The weir equation is applied for all 5 return periods and furnished in Table 1.

**Table 1:** Changes in flood depth in Velachery for different return periods.

Return period (year)	Peak discharge (m^3^/s)	Head over the weir (m)		The decrease in flood depth (m)
		Existing channel width = 120 m	Proposed channel width = 200 m	
2	372.4	1.45	1.03	0.42
5	566.2	1.92	1.37	0.55
10	674.2	2.16	1.54	0.62
50	933.8	2.68	1.91	0.77
100	1063.7	2.93	2.08	0.85

From the Table 1, peak discharge has increased about 185% from 2 year to 100 year return period. To reduce the flood depth, the width of the weir can be increased. Hence, it is suggested to increase the width of weir from 120 m to 200 m. Thus, it can significantly reduce the flood depth by increasing the width of Okkiyam Maduvu in order to cope with extreme events. A minimum width of 200 m in the downstream channel is required to ensure the free flow of flood water. The head over the weir for 2 year return period is 1.45 m when the channel width is 120 m and when the channel width is increased to 200 m the head is decreased by 42 cm. For 100 year return period, flood depth would decrease by 85 cm when the width of the channel is increased from 120 m to 200 m.

## Conclusion and Recommendations

IDF curves for observed rainfall indicate that around 162% increase in the intensity of rainfall from 2 year to 100 year return period. Comparison between observed and projected IDF curves under changing climate scenario shows that there is an increase of 12% intensity for 2 year return period rainfall and 87% increase for 100 year return period. The assessment of Velachery drainage system response to storm events of various return periods under present and future climate change scenarios idendifies the vulnerable hot-spots including critically flooded channels and outflows. The highest peak discharge is observed in Outfall 1 which is located near to Velachery lake and joins with Pallikarani marshland. Maximum flooding is noticed at junction VB-179 which is located very near to Velachery Lake. Out of 234 nodes/junctions, 9 nodes are identified as hotspots and flooding noticed under all storm events. These nodes are inundated for nearly 24 hours. Proper maintenance should be taken routinely inorder to make a free way to drain out the flow and to avoid the blockages in storm drains. It will help to reduce the detention period. Construction of the new road should not obstruct the passage or the inlet of the storm drains. Storm drains constructed in some places should be redesigned to cope with future flood events. Increasing size of the Okkiam Maduvu weir width from 120 m to 200 m, will reduce the depth of the flow over the weir from 3.43 m to 2.44 m for 100 yr return period rainfall thus reducing the backwater effect. At Okkiam Maduvu, minimum width of 200 m in the downstream channel is essential to ensure the free flow of flood water. The carrying capacity of the channel may be improved by removing vegetation and blockages and lining with stone pitching.

This study provides a holistic approach to study flood management through integrating the climate knowledge with the flood frequency of Chennai. The present study undertakes a hybrid approach by combining climate scenarios with urban storm drainage network to address the pluvial flooding problem of the study area. There is a good agreement with the data analyzed on climate extremities and frequency of urban flooding. The successful incorporation of the scientifically reliable climate information in hydrologic and hydraulic models demonstrates the versatility of this technique for site specific flood management studies. Moreover, in this study, hydrologic and hydraulic modelling are well supported with strong field data and actual flow measurements. This work uses high resolution DEM (10 m resolution) procured from National Remote Sensing Center. However it is desirable to have fine resolution DEM preferably with sub meter accuracy which can be opted better results. Besides, incorporating real time flood forecasting with Low Impact Development (LID) practices such as permeable pavements, rain gardens, green roofs, street planters, rain barrels, infiltration trenches and vegetative swales in Hydrologic modelling in future studies will provide more accurate and effective flood management system.

### Methodology

The critical rainfall events and different physical characteristics are used to describe the response of Velachery drainage system. The present study uses GCMs for future climate scenarios, HEC-HMS model for watershed runoff and SWMM for storm drainage network to estimate the discharge and flooded areas of Velachery zone and to suggest mitigation measures.

### Climate change scenarios

The past rainfall data for the period (rainfall 1975–2015) were procured from Regional Meteorological Centre (RMC), India Meteorological Department (IMD), Chennai. The occurrence of extreme events were calculated based on IMD norms. The extremely heavy rainfall event (>244.5 mm/day) and very heavy rainfall event (>124.5 mm/day) for the period of 1975–2015 were calculated. Potential anthropogenic climate change scenarios of future and their underlying driving forces are always an important component of any impact assessments. The driving forces are well captured and updated in IPCC’s continuous assessment reports and in Global Climate Models (GCM). The recent IPCC AR5 adopted Representative Concentration Pathways (RCP) to illustrate the concentration of future anthropogenic greenhouse gas (GHG) emissions via., RCP 2.6, RCP 4.5, RCP 6.0 and RCP 8.5. RCP 2.6 is peak scenario and represents very low greenhouse gas concentration levels. RCP 4.5 is balanced mitigation scenario or stabilization scenario where total radiative forcing is stabilized before the year 2100 by the employment of a range of technologies and strategies for reducing greenhouse gas emissions. RCP 6.0 is stabilization scenario where total radiative forcing is stabilized before 2100 without overshoot employment of a range of technologies and strategies for reducing greenhouse gas emissions. RCP 8.5 is business-as-usual scenario leading to high greenhouse gas concentration levels^10,11^. Global Climate Model (GCM) data from CMIP5 (Coupled Model Intercomparison Project Phase 5) dataset of IPCC AR5^12^ for RCP 4.5 scenario were used inthis study to project the future climate change scenario for the period 2015–2085^13,14^. The GCM models were statistically downloaded from http://ccafs-climate.org/. The GCM models cesm1_cam5, mpi_esm_mr, ncar_ccsm4, bnu_esm were used to project future climate scenarios of the study area and to generate the IDF curves. These GCM data were developed by various pioneer institutions in different countries. bnu_esm was developed by Beijing Normal University, China. cesm1_cam5 and ncar_ccsm4 were developed by National Center for Atmospheric Research, USA. Max Planck Institute for Meteorology, Germany developed the model mpi_esm_mr.

### Generation of Intensity Duration Frequency (IDF) curves

The daily rainfall data of past and future climate scenarios were used to develop the IDF curves^15^. IDF curves were developed using observed data and GCM models for 5 different return periods of 2, 5, 10, 50, 100 years. From daily rainfall data, hourly rainfall data were calculated using IMD’s empirical reduction formula^16^. In this study, the following empirical equation is used to estimate short duration rainfall.

1 $${{\rm{P}}}_{{\rm{t}}}={{\rm{P}}}_{24}\sqrt[3]{\frac{{\rm{t}}}{24},}$$

where P_t_ is required rainfall depth in mm at t-h duration, P_24_ is daily rainfall in mm, t is duration of rainfall in h. Hourly rainfalls of various duration like 1-h, 2-h, 6-h, 12-h, 24-h rainfall values were calculated from annual maximum values. The mean and standard deviation for the data for different durations is calculated. Mean hourly rainfall of Meenambakkam station for 1 h, 2 h, 6 h, 12 h and 24 h are 54.77 mm, 34.50 mm, 16.59 mm, 10.45 mm and 6.58 likewise standard deviation for that durations are 24.99 mm, 15.75 mm, 7.57 mm, 4.77 mm and 3.00 mm. Gumbel’s Extreme Value distribution is most commonly used for IDF relationships and used here to fit probability distribution. K_T_ values are calculated as −0.164, 0.719, 1.305, 2.592 and 3.137 using Eq. 2 for the return periods 2 yr, 5 yr, 10 yr, 50 yr and 100 yr using Gumbel’s distribution^17^.

2 $${{\rm{K}}}_{{\rm{T}}}=-\frac{\sqrt{6}}{{\rm{\pi }}}\{0.5772+\,\mathrm{ln}[\mathrm{ln}(\frac{{\rm{T}}}{{\rm{T}}-1})]\},$$

Then, rainfall depth or intensity was calculated for a given return period. The formula for getting rainfall intensity using frequency factor is given in Eq. 3.

3 $${{\rm{X}}}_{{\rm{T}}}=\bar{{\rm{X}}}+{{\rm{K}}}_{{\rm{T}}}\,S,$$

where, *X*_*T*_ is rainfall intensity at given return period, $\bar{X}$ is mean of particular time, *S* is standard deviation, *K*_*T*_ is frequency factor.

### Base map and Digital Elevation Model (DEM)

CartoDEM data of 10 m resolution was procured from National Remote Sensing Centre (NRSC), Hyderabad. Nearly 4 tiles which covered the study area were obtained. All the procured tiles were merged with a mosaic option in ERDAS IMAGINE and geo-corrected in ArcGIS 10.1 software. Velachery ward maps (ward nos. 177, 178 and 179) were collected from Corporation of Chennai. Using ward maps as base, the administrative boundary layer of the study area was digitized using 1:50000 scale toposheet (66 D1 & D5) which were from Survey of India. This map was used to clip study area from the DEM. Both DEM and boundary map were used to delineate the sub-catchments using ArcGIS. Terrain characteristics like slope were derived from DEM and used for further hydrological modelling. The impervious areas were marked using Google Earth and percentage imperviousness was calculated.

### Differential Geographic Positioning System (DGPS) survey

**Figure 7:**
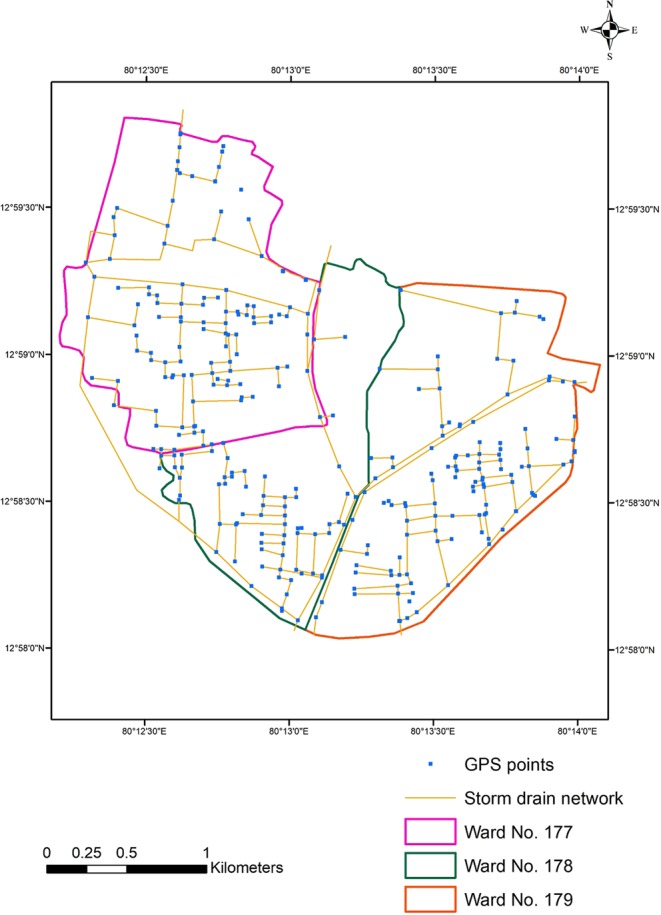
Delineation of storm drainage network in wards 177, 178 and 179 of Velachery in Chennai Metropolitan Area [ indicates DGPS Survey points].

Existing storm water drainage network details of Velachery ward nos 177, 178 and 179 were collected from Corporation of Chennai. Based on these collected details from Chennai Corporation, DGPS survey was carried out in Velachery using Leica DGPS (Fig. 7). Totally 319 points were measured and the invert levels of the nodes in the storm drain network were derived from the DGPS survey.

### Inundation modelling using SWMM

Several mathematical models are widely used to model the dynamics of rainfall-runoff and flood generation processes such as MIKE URBAN, HEC-HMS, SWMM, DRAINS, MOUSE, Infowork RS, HSPF, DR3M, STORM etc^18^. These urban storm water flow models may prove to be immensely beneficial in ascertaining the effects of various storm water management strategies and evaluating the storm readiness and climate resilience of the cities. Storm Water Management Model (SWMM), developed by the US Environmental Protection Agency (EPA), is a comprehensive computer model for simulating hydrological and hydraulic processes of an urban watershed^19–21^. It is widely used for modelling storm water quantity and quality in an urban environment^1,22^. SWMM has been packaged along with user-friendly interfaces by commercial software developers such as XP-SWMM, MIKE-SWMM, MIKE-URBAN, PC SWMM, thereby resulting into its wider dissemination^18,23,24^. SWMM requires detailed information characterizing urban catchments and the underlying storm water drainage infrastructure. The input to the models comprises of various physical and hydrological parameters representing urban catchment^25^. The surface runoff in a sub-catchment is routed fully to enter the junction and transported through the storm sewer conduits to the area outlet, so there is only pipeline runoff flow and no surface runoff routing is considered. The model has been used for various urban catchments in India particularly for a single watershed under uncalibrated condition^6,20,26^.

SWMM is a dynamic rainfall-runoff simulation model and can be used for either single event or long-term (continuous) simulation of runoff quantity from urban areas^6^. The hydrological processes were set up with creating parameters such as rain gauge, sub-catchment, node, link and outfall. The sub-catchment delineation was done with ArcSWAT tool in ArcGIS using CartoDEM of 10 m resolution with the help of a base map prepared. The storm water drainage network was created by including the junction nodes and the conduits. Each sub-catchment was joined with corresponding nodes. The design parameters of the junctions include junction ID, invert level and diameter. The design parameters of the conduit include conduit ID, shape, inlet node, outlet node, length, maximum depth, roughness, and flow through pipe and loss coefficient. The nodes were linked by conduits and in a total of 238 nodes and 4 outfalls and 240 links were created. Links represent the channel and nodes indicate junction or change in direction (Fig. 4).

The details of the drains such as size and length were collected from Corporation of Chennai Zonal office, Adyar. Pervious and impervious areas were digitized with Google Earth, then percentage imperviousness was calculated. Nearly 88.5% area is impervious. Normally the storm drains are rectangular concrete channels with rough forms, so the Manning’s roughness coefficient (n) was taken to be 0.015. Green-Ampt infiltration method was selected with suction head 8.27 mm, conductivity 0.04 mm/hr and an initial deficit of 0.2 mm/hr. Dynamic Wave Routing method was adopted to solve the Saint-Venant flow equations.

The number of nodes flooded more than 20 hours were identified as vulnerable hot spots. To visualize the hotspots geo-spatially, vulnerability map was generated using the image procured from National Remote Sensing Centre (NRSC), Hyderabad Govt. of India as a base, the hot spots identified through modelling were then overlay using ArcGIS 10.1 software.

### Runoff modelling using HEC-HMS

The runoff from Velachery zone discharges into Pallikaranai marshland. Pallikaraani marshland receives run-off from its adjoining catchment including Velachery, Madipakkam, and Perumbakkam etc., the storm water then passes through the Okkiyam Maduvu channel. Okkiyam Maduvu is a 2.8 kilometers long water channel originates as a narrow canal from the Pallikaranai marshland and drains into the Buckingham canal which flows south and enters the Kovalam estuary as shown in Fig. 1. However, modelling of urban drainage system needs integrated modelling while considering flood mitigation measures for Velachery, peak discharge at Okkiyam Maduvu, is essential.

However, modelling of urban drainage system needs combined modelling and is done successfully by integrating SWMM with HEC-HMS^27^. HEC-HMS was developed by U.S. Army Corps of Engineers designed to simulate the rainfall-runoff processes of dendritic watershed systems^28,29^. The inputs to the model include land use information, hydrologic soil group and rainfall. Physical characteristics such as the river length and slope, the sub-basin centroid location and elevation, the longest flow path for each sub-basin, and the length along the stream path are extracted using ArcGIS^30^. The impact of climate change on the basin hydrology is examined by comparing the present and future streamflow using HEC-HMS. The simulations are carried out using statistically downscaled Global Climate Models (GCM) models to determine the future climate impacts on the flow^31,32^.

Hence HEC-HMS model was used for runoff simulation at Okkiyam Maduvu and then calculate the carrying capacity of the channel to drain the storm water into Buckingham canal. The watershed delineation for Okkiyam Maduvu was done using CartoDEM. The basin model created using HEC-GeoHMS was imported to HEC-HMS model. The land use data, hydrologic soil group and curve numbers were given as inputs for HEC-HMS model. The peak discharge calculated from the model simulation was used to evaluate the carrying capacity of Okkiyam Maduvu and possible mitigation measures were suggested. Based on this peak discharge, adequate width required to reduce the flood depth through Okkiyam Maduvu was calculated using the formula for rectangular weir (Eq. 4).

4 $${\rm{Q}}={{\rm{c}}}_{{\rm{d}}}\frac{2}{3}{\rm{b}}(\sqrt{2{\rm{g}}}){{\rm{H}}}^{\frac{3}{2}},$$

where Q is Flow rate (m^3^/s), C_d_ is discharge coefficient (0.6), b is width of the weir (m), g is acceleration due to gravity (9.81 m/s^2^) and H is height of water above the crest of the weir (m).
